# Molecular Evolution of *PvMSP3α* Block II in *Plasmodium vivax* from Diverse Geographic Origins

**DOI:** 10.1371/journal.pone.0135396

**Published:** 2015-08-12

**Authors:** Bhavna Gupta, B. P. Niranjan Reddy, Qi Fan, Guiyun Yan, Jeeraphat Sirichaisinthop, Jetsumon Sattabongkot, Ananias A. Escalante, Liwang Cui

**Affiliations:** 1 Department of Entomology, Pennsylvania State University, University Park, PA 16802, United States of America; 2 Dalian Institute of Biotechnology, Dalian, Liaoning, China; 3 Program in Public Health, University of California, Irvine, CA 92697, United States of America; 4 Vector Borne Disease Training Center, Pra Budhabat, Saraburi 18120, Thailand; 5 Mahidol Vivax Research Unit, Faculty of Tropical Medicine, Mahidol University, Bangkok, 10400 Thailand; 6 Institute for Genomics and Evolutionary Medicine, Temple University, Philadelphia, PA, United States of America; Centro de Pesquisa Rene Rachou/Fundação Oswaldo Cruz (Fiocruz-Minas), BRAZIL

## Abstract

Block II of *Plasmodium vivax* merozoite surface protein 3α (*PvMSP3α*) is conserved and has been proposed as a potential candidate for a malaria vaccine. The present study aimed to compare sequence diversity in *PvMSP3a* block II at a local microgeographic scale in a village as well as from larger geographic regions (countries and worldwide). Blood samples were collected from asymptomatic carriers of *P*. *vivax* in a village at the western border of Thailand and *PvMSP3α* was amplified and sequenced. For population genetic analysis, 237 *PvMSP3α* block II sequences from eleven *P*. *vivax* endemic countries were analyzed. *PvMSP3α* sequences from 20 village-level samples revealed two length variant types with one type containing a large deletion in block I. In contrast, block II was relatively conserved; especially, some non-synonymous mutations were extensively shared among 11 parasite populations. However, the majority of the low-frequency synonymous variations were population specific. The conserved pattern of nucleotide diversity in block II sequences was probably due to functional/structural constraints, which were further supported by the tests of neutrality. Notably, a small region in block II that encodes a predicted B cell epitope was highly polymorphic and showed signs of balancing selection, signifying that this region might be influenced by the immune selection and may serve as a starting point for designing multi-antigen/stage epitope based vaccines against this parasite.

## Introduction

Vaccine is a long-term hope to combat malaria—a major infectious disease responsible for more than half a million deaths annually around the world. The alarming signals of artemisinin resistant parasites seemingly to follow the same path initially laid down by chloroquine-resistant parasites across international borders in Southeast Asia further urge the development of vaccines against malaria [1, 2]. Vaccine research has been largely focused on *Plasmodium falciparum*—a species responsible for the majority of malaria-related deaths. However, vaccine research for *P*. *vivax* has trailed far behind [3, 4]. Yet, *P*. *vivax* is the most widespread human malaria parasite and it causes 50–70 million infections annually [5]. This co-called ‘benign tertian’ malaria parasite has been increasingly recognized as the cause of significant morbidity and mortality. The changing malaria epidemiology worldwide with increasing proportions of *P*. *vivax* malaria further highlights the difficulty for controlling this parasite and emphasizes the need to develop integrated control strategies including vaccine for this parasite [6].

Several antigens have been proposed as potential vaccine candidates for *P*. *falciparum* [7], and their orthologs in *P*. *vivax* (*PvAMA*-1, *PvMSP*-1, *PvDBP*, *PvCSP*, *PvMSP3α*, etc.) have also been characterized. Antigenic diversity in these genes [8–14] has significantly hindered the progress in vaccine research [15–17], since multiple antigenic alleles could evade vaccine-induced, allele-specific immunity. In contrast, antigens with low variability or the conserved functional regions of polymorphic antigens are attractive vaccine targets [17], as these regions are assumed to be under functional constraints and possibly have slower evolutionary mechanisms. This approach has been used for MSP3-LSP and GMZ2 vaccines which include conserved C-terminal region of the *P*. *falciparum* merozoite surface protein 3 (*PfMSP3*) gene [18, 19]. Furthermore, various genomics and proteomics approaches are being exploited to identify such conserved regions to overcome the challenges imposed by genetic variations [20].

*MSP3* in *P*. *vivax* is a family of 11 members with a complex evolutionary history [21, 22]. Two of the loci, *MSP3α* and *3β*, have been widely used as population genetic markers for typing *P*. *vivax* isolates based on polymorphisms depicted by PCR/RFLP analysis [10, 23–25]. Previous studies analyzing *PvMSP3α* gene sequences have observed differential pattern of diversity across different domains of the gene [10, 26, 27]. *PvMSP3α* is composed of an N-terminal signal sequence, a central alanine rich region and an acidic C-terminus. The alanine rich repeat region of *PvMSP3α* encodes block I (residues 104–396) and block II (434–687). Block II has been shown to be relatively conserved with non-random variations clustered in two structural motifs; motif I from amino acid position 533 to 538 and motif II from 580 to 587 [26, 27]. Interestingly, the variations within each motif are tightly linked that have generated dimorphic alleles for each motif (motif I: MSELEK/LSKLEE and motif II: TAANVVKD/KEATAAKL). All of these alleles have been found equally prevalent in natural *P*. *vivax* populations [10, 24, 26, 27]. Based on this peculiar pattern of variation, block II has generated considerable interest as a potential vaccine candidate. Block II is also known to elicit a pronounced antibody response against clinical malaria infections reported from Papua New Guinea [28] and Brazil Amazon [29]. In fact one of the studies suggested that block II specific antibodies compared to other regions of the gene are more responsive to high density natural infections [28]. All these features point to block II as a potential vaccine candidate or target for sero-epidemiology studies in *P*. *vivax*.

A conserved pattern of variation in an antigenic sequence has been widely associated with purifying (negative) selection that can be the result of either structural constraints or strong immune directional selection [30, 31]. On the contrary, high diversity has been usually associated with balancing selection by the immune system [32, 33] but alternatively could be the result of relaxation. Since antigenic diversity is generally influenced by local endemic settings, comparative analysis of the gene in diverse population backgrounds is more informative. Genetic diversity in block II of *PvMSP3α* has been previously characterized only in a few laboratory adapted strains [26], and limited clinical samples from Thailand [10, 34] and Venezuela [24, 27]. Thus, this study aimed to define the patterns of variation in *PvMSP3α* block II in samples from a small village (local diversity) compared with the larger geographic structures. By studying the extent and distribution of polymorphisms in *PvMSP3α* block II among *P*. *vivax* samples from 11 countries, we hope to understand the evolutionary mechanism underlying the variation patterns. Genetic diversity in block II preferentially revealed large number of rare alleles, and high frequency variants were restricted to specific genetic regions. The prominent allelic forms of block II were extensively shared by diverse *P*. *vivax* populations.

## Material and Methods

### Study area and sampling

**Fig 1:**
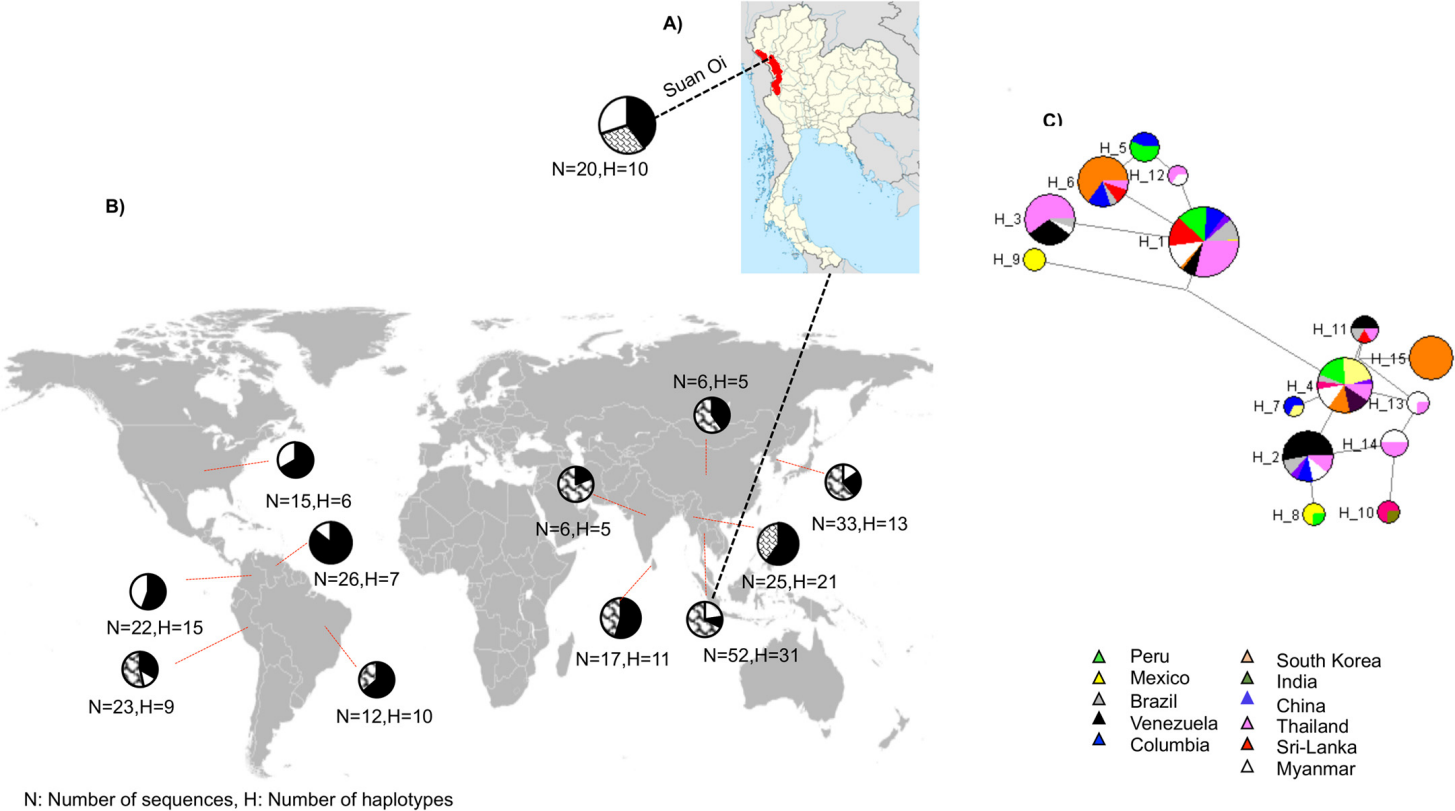
Location of sampling sites with *PvMSP3α* block II allelic forms. **(A)** The location of Suan Oi village at the western border of Thailand. **(B)** A map showing the distribution of block II allelic forms across eleven countries. The black portion of the pie chart indicates shared alleles (with one or more populations), while the white portion shows population specific alleles. Population specific alleles that were observed only once (singletons) are shown as pattern-filled portion in the pie chart. **(C)** Haplotype network constructed from block II alleles generated using only non-synonymous variations that were seen in more than two isolates. Both the maps of the world and Thailand are taken from Wikimedia Commons.

The present study was conducted in a small village Suan Oi in Tha Song Yang district of Tak province located at western border of Thailand (Fig 1A), which is known to contribute highest number of malaria cases in the country [35]. Malaria in this region is hypo-endemic and seasonal [35, 36]. *P*. *falciparum* and *P*. *vivax* infections are more prevalent and notably, we have reported a large number of asymptomatic infections (only detected by expert microscopists and by PCR assay) in that area recently [35, 36]. Asymptomatic infections usually remain undetected by passive case detection by hospitals and clinics [35], thus present study conducted mass blood surveys in Suan Oi village between June 2011 and June 2013 to determine the magnitude of *P*. *vivax* infections at a micro-geographic scale.

Finger-pricked blood samples were obtained from all the residents of Suan Oi during mass blood surveys. The presence of malaria parasites in some of the blood samples from participants were confirmed by microscopic examination of Giemsa-stained blood films and by PCR [36]. Genomic DNA was extracted from dried blood spots on Whatman filter paper using a QiaAmp DNA Mini Kit (Qiagen, Germany). *Plasmodium* species identification was carried out by species-specific rDNA based primers following method described in Snounou et al., 1993. Twenty four samples showing single *P*. *vivax* infections were included in sequencing analysis.

### Ethics statement

Written informed consent was obtained from the participants or guardians. This study was approved by the Institutional Review Boards of Pennsylvania State University and Thai Ministry of Public Health.

### Sequencing of *PvMSP3α* gene

*PvMSP3α* in *P*. *vivax* samples was amplified using primers described previously [10]. Amplified fragments were visualized on 1.5% agarose gel for approximate size estimation. PCR amplified fragments were further purified using the High Pure PCR cleanup microkit (Roche) and sequenced in both directions using BigDye Terminator v3.1. DNA sequences obtained were assembled using Lasergene software (DNASTAR) with manual editing, and aligned with the Sal I reference gene sequence (PVX_097720) using ClustalW. The sequences corresponding to block II region of *PvMSP3α* present in all samples were extracted for analysis.

### Data collection

*PvMSP3α* block II sequences generated in the present study were compared and analyzed together with the sequences retrieved from GenBank (http://www.ncbi.nlm.nih.gov/genbank/) and Plasmodb (http://plasmodb.org/plasmo/) database. Totally, 237 sequences were derived from 11 parasite populations, which included 52 samples from Thailand (including 20 samples from the present study [10, 34]; 25 from Myanmar [9], 6 from India, 6 from China, 25 from South Korea [37], 17 from Sri Lanka [38], 12 from Brazil, 22 from Colombia, 23 from Peru, 26 from Venezuela [27] and 15 from Mexico (S1 Table). Excluding indels and multiple alleles, the 695 bp region encoding block II (nucleotide 2078 to 2773) from all 237 samples were used for analysis.

### Population genetic analysis

Within population, polymorphism was quantified by total number of segregating sites and haplotypes. Genetic diversity was measured by average pairwise nucleotide diversity (θπ) and haplotype diversity (Hd) [39]. Local diversity measures estimated for each population were compared with overall worldwide diversity. Genetic differentiation between populations was estimated using Wright’s F_ST_−a measure of fixation index [40] and the statistical significance of the F_ST_ values was tested through 1000 random permutations. All the above analyses were performed using DnaSP v5 software [41].

Phylogeographic clustering of the isolates was evaluated by Maximum Likelihood (ML) tree in MEGA6 [42] using Tamura and Nei’s model of nucleotide substitution. Support for individual nodes was obtained by performing 500 bootstrap replicates. In order to visualize the distribution of immunologically relevant polymorphisms across populations, haplotypes were constructed from non-synonymous SNPs that were observed in more than two isolates (excluding singletons and doubletons), as singletons and low frequency alleles are not generally considered informative for vaccine design [43]. Haplotype network was drawn by NETWORK (fluxus-engineering.com) using the median joining algorithm [44].

To examine departure from neutrality, we estimated the numbers of synonymous substitutions per synonymous site (dS) and of nonsynonymous substitutions per nonsynonymous site (dN) using the Nei and Gojobori method [45] as implemented in MEGA6. Significance of the difference between dN and dS was estimated with a Z-test of selection. A dN significantly higher than dS is consistent with positive selection, while dS higher than dN is expected under purifying selection. We also used Tajima’s D [46] and Fu and Li’s F* [47] frequency-based tests of neutrality implemented in DnaSP v5 to examine departure from neutrality. Tajima’s D test compares average pairwise nucleotide diversity (θπ) with the standardized number of polymorphic sites per site (θS), whereas Fu and Li’s F* tests excess or lack of singletons by comparing number of singletons and the average number of nucleotide differences between two sequences. Significantly positive values of these tests suggest a recent population bottleneck or balancing selection, whereas negative values indicate population growth or directional selection.

We used an array of methods to detect recombination signals viz; RDP, MaxChi, GENECONV, GARD and minimum number of recombination events (*Rm*) according to the four-gamete test by Hudson & Kaplan [48]. We used the RDP3 package [49] for RDP [50], MaxChi [51], and GENECONV [52]. These methods are designed to detect recombination breakpoints. RDP and MaxChi are used in sliding window analysis, whereas GENECONV scans for long regions of identity between sequences. We also used another tree based method of recombination detection, Genetic Algorithm for Recombination Detection (GARD) [53] implemented in Datamonkey (datamonkey.org) [54], which identifies recombination breakpoints by searching for significant change in the nodes of the tree constructed from all possible partitions. Recombination rate (*ρ*) and mutation rate (*θ*) were calculated using LDhat package [55].

## Results

### Polymorphisms in *PvMSP3α* gene in the western Thai village

The *PvMSP3α* gene displays enormous genetic diversity in *P*. *vivax* populations and consequently has been used as a molecular marker for differentiating field parasite strains [24]. To investigate the genetic diversity of *PvMSP3α* gene on a microgeographic scale, we collected *P*. *vivax* samples from asymptomatic carriers in a small village Suan Oi (~500 residents) in western Thailand (Fig 1A) during mass blood surveys conducted in this area. The *PvMSP3α* gene was successfully amplified in 22 of 24 *P*. *vivax* samples. Two PCR length variant types A (1.9 Kb) and C (1.1 Kb) were observed, whereas type B (1.5 Kb) was not observed in the tested samples [34]. Two of the samples produced two bands, suggesting of mixed strain infections.

In order to determine the details of sequence diversity of *PvMSP3α*, PCR products of 20 samples were sequenced and aligned with the Sal I reference gene. PCR fragment type C contained a ~750 bp deletion in block I, whereas block II was relatively conserved in all 20 samples. *PvMSP3α* block II in the 20 samples had 28 single nucleotide polymorphisms (SNPs), 24 of which were parsimony informative sites (SNPs observed in more than one sequence). Among these SNPs, 15 were non-synonymous mutations which changed 15 amino acids (12 as parsimony informative). Nucleotide diversity of block II was 0.013 and haplotype diversity was 0.800 with 10 haplotypes/allelic forms of block II.

### Genetic diversity of *PvMSP3α* in Thailand

We compared the genetic diversity of the *PvMSP3α* block II sequences from the Suan Oi isolates with 32 publically available *PvMSP3α* sequences from Thailand. A total of 85 mutations were observed in 32 sequences, of which 59 were singletons. These sequences differed from the Suan Oi samples in the excess number of singletons. While singletons could be potential consequences of sequencing errors or population expansions [56], low frequency alleles are not generally considered very informative in vaccine design [43]. Twelve parsimony informative amino acid changes were observed in the 32 sequences, of which 10 were shared with the Suan Oi samples, indicating that these high-frequency variants are commonly present in samples from diverse regions of Thailand and these parasites persisted during the last ten years. When all the 52 samples from Thailand were analyzed together (hereafter named as Thai samples), a total of 87 SNPs were observed with 50 amino acid changes, of which only 14 were parsimony informative. As expected from previous studies, nine of the 14 amino acid changes were clustered in two structural motifs (motif I: 533 to 538 and motif II: 580 to 587). The SNPs in each motif are tightly linked and formed two major alleles for each motif (motif I: MSELEK/LSKLEE and motif II: TAANVVKD/KEATAAKL) [34].

### Worldwide genetic diversity in *PvMSP3α* block II

**Fig 2:**
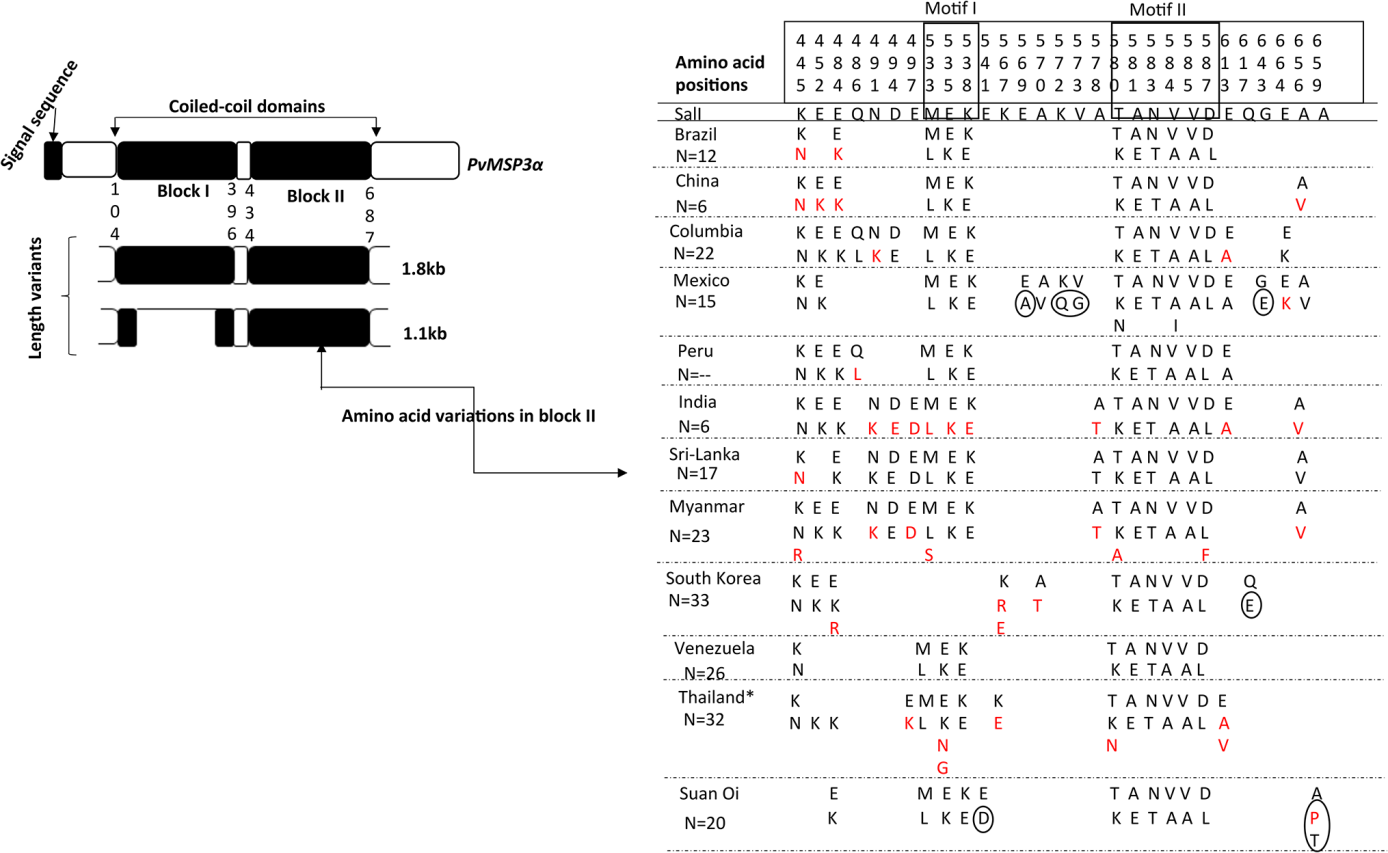
Polymorphism and its pattern in *PvMSP3α* and block II. Left panel shows a schematic representation of different domains of *PvMSP3*α and two genotypes of the locus observed after PCR amplification of the Suan Oi isolates. Right panel shows distribution of amino acid substitutions (excluding singletons in all 237 sequences) in each country. Amino acid positions are numbered corresponding to Salvador I reference strain and the changes in two structural motifs are boxed. Mutations that are observed only once in particular country are highlighted in red and country-specific mutations are circled.

We further investigated the worldwide extent of genetic diversity in a total of 237 sequences of *PvMSP3α* block II from 11 parasite populations. The sequences included 52 obtained from Thailand and 185 of worldwide isolates retrieved from the GenBank and Plasmodb database (S1 Table). The parasite populations of the 237 samples included 139 sequences from Asian countries (China, India, Sri Lanka, Myanmar, South Korea, and Thailand) and 98 from America (Brazil, Colombia, Mexico, Peru, and Venezuela) (Fig 1B). These sequences contained 158 SNPs ranging from 24–86 in each country, which resulted in 76 amino acid changes (Table 1). Asian samples showed a relatively higher number of singletons compared to American populations (Table 1). However, the high-frequency non-synonymous mutations were extensively shared by all populations from Asia and America. Twenty nine of the total amino acid changes were parsimony informative, of which only 13 had a frequency of more than 5% and 10 of them were present in two aforementioned structural motifs (Fig 2). A similar pattern of amino acid variations was observed in each population. Population-specific polymorphisms were mostly singletons and synonymous in nature.

**Table 1:** Single nucleotide polymorphisms and summary statistics of *PvMSP3α* block II in different geographical regions.

Population	No. of isolates	SNPs	Singletons	Amino acid changes (Parsimony informative)	Haplotypes	θπ	Haplotype diversity	Tajima’s D	Fu & Li’s F
**Sua Oi**	**20**	**28**	4	**12**	**10**	**0.013**	**0.800**	**0.7558**	**0.797**
Thailand^#^	52	87	60	14	31	0.017	0.947	-1.3265	-3.835**
India	6	39	25	7	5	0.023	0.933	-0.4033	-0.4219
China	6	27	6	9	5	0.019	0.933	0.7700	1.0298
Myanmar	25	60	33	13	21	0.021	0.977	-0.2238	-1.5189
Sri Lanka	17	36	9	15	11	0.017	0.926	0.4825	0.4395
South Korea	33	36	14	10	13	0.016	0.816	1.1073	-0.3151
Brazil	12	29	8	9	10	0.015	0.955	0.4974	0.4652
Mexico	15	38	5	17	6	0.020	0.819	0.9332	1.1185
Peru	23	29	1	13	9	0.016	0.897	1.7677*	1.8257**
Colombia	22	35	3	15	15	0.016	0.961	0.6206	1.1887
Venezuela	26	24	0	10	7	0.015	0.818	2.4208**	2.2213**
**World-wide**	**237**	**158**	**108**	**29**	**100**	**0.019**	**0.972**	-1.5136*	-6.5208**

**Fig 3:**
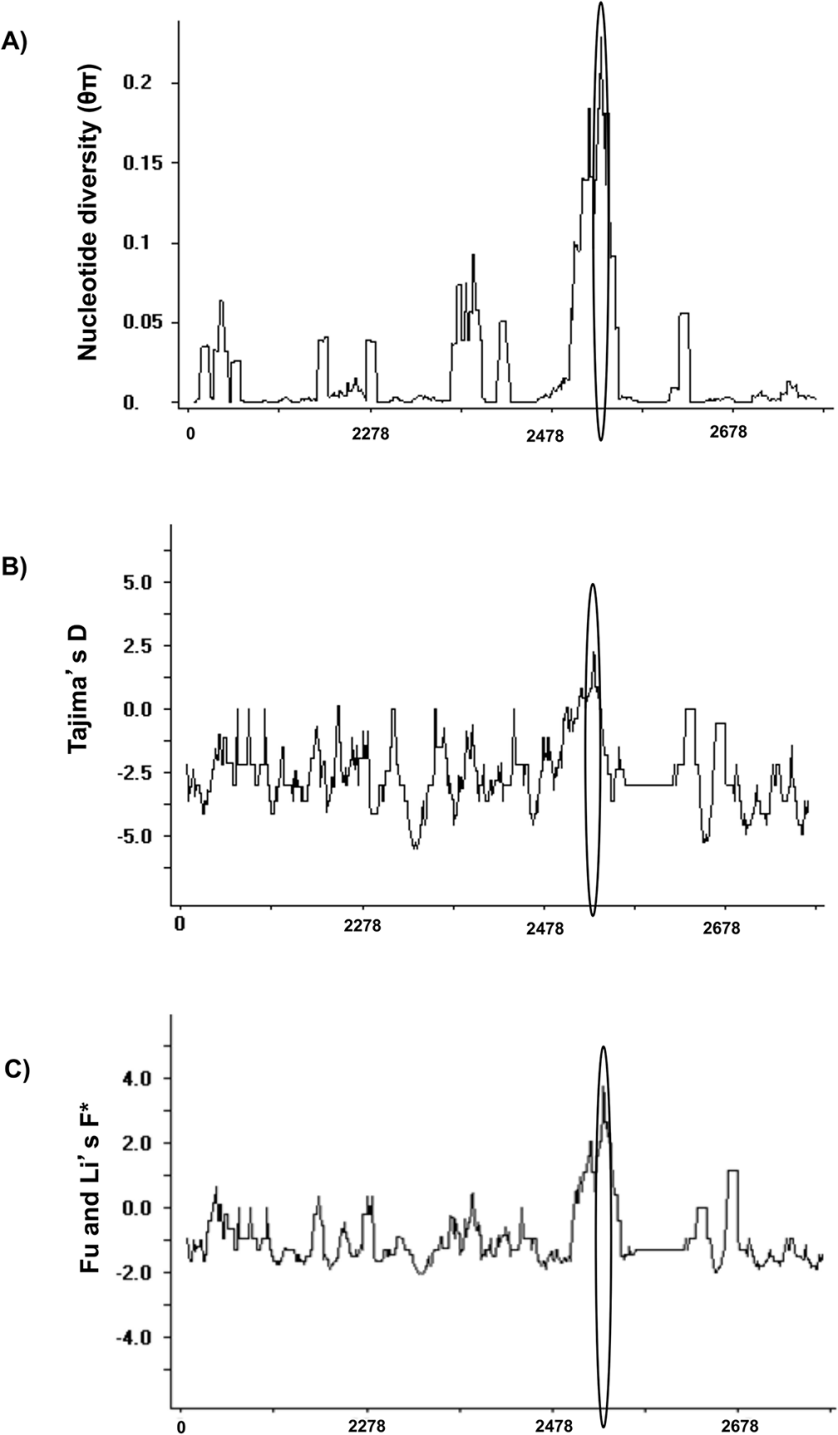
Sliding window plot analysis of nucleotide diversity and tests of selection on *PvMSP3α* block II in worldwide sequences. (**A)** Average pairwise nucleotide diversity (θπ). (**B)** Tajima’s D values. (**C)** Fu & Li’s F* test values. A window size of 11 and step size of 1 bp were used. The region with the highest peak of significant values is circled.

Nucleotide diversity was 0.019 in worldwide samples, ranging from 0.015 to 0.023 in 11 parasite populations (Table 1). θπ was relatively high in India (0.023) and lowest in Brazil and Venezuela (0.015). A sliding window plot of θπ revealed a peak (0.069) at nucleotide positions 2477–2577 (positions corresponding to the Sal I sequence) (Fig 3A), and a similar trend was observed in all the populations. Again, this region encodes structural motif II, where 6 of 13 high-frequency amino acid variants (minor allele frequency >5%) were identified.

The 237 sequences had a total of 100 allelic forms of block II (from 5 in India and China to 31 in Thai samples) with an overall allelic diversity of 0.9724 (0.816–0.977 country-wise). Fifteen block II alleles were shared between populations and 85 were population specific. Both shared and population-specific alleles were observed in each population. Interestingly, all the population-specific alleles were singletons in five out of the 11 populations (Fig 1B). Population specific alleles are not the preferred choice for formulating vaccines aiming to control pathogens worldwide. Haplotype network constructed from the block II allelic forms observed in more than two isolates revealed 15 alleles, of which 6 were shared among four or more populations (Fig 1C). Highly frequent block II allelic forms were shared by diverse population samples from Asia and America (Fig 1C).

### Population differentiation

Genetic differentiation between worldwide populations estimated using F_ST_ showed a modest genetic structure (0.083), which means population genetic differentiation accounting for only 8% of the total variations in the gene. The highest degree of population differentiation was observed between Thail and Mexico (F_ST_ = 0.2559, P<0.001) and the lowest between Mexico and China (F_ST_ = -0.0025, P>0.05; Table 2), suggesting that geographic distance is not significantly responsible for genetic differentiation. Moreover, F_ST_ values did not correlate with the geographic distance between the populations (Spearman correlation coefficient = -0.0336, *P* = 0.8).

**Table 2:** Pairwise F_ST_ estimates for 11 *Plasmodium vivax* endemic countries using *PvMSP3α* block II sequences.

Populations	Brazil	China	Colombia	India	Mexico	Peru	Sri-Lanka	Myanmar	South Korea	Venezuela
China	0.0166									
Colombia	-0.0126	0.1035								
India	0.1046	-0.1018	**0.1323**							
Mexico	**0.1734**	-0.0025	**0.2271**	-0.0041						
Peru	-0.0051	0.0233	-0.0083	0.0329	**0.1542**					
Sri-Lanka	-0.0314	0.0610	-0.0032	0.1180	**0.1975**	0.0308				
Myanmar	0.0363	-0.0951	0.1114	0.0005	**0.0948**	0.0567	0.0717			
South Korea	**0.1653**	0.0588	**0.1344**	0.0363	**0.1902**	**0.0801**	**0.1472**	**0.1140**		
Venezuela	**0.0942**	-0.0699	**0.2087**	**0.0435**	**0.1083**	**0.1402**	**0.1606**	0.0002	**0.2297**	
Thailand	-0.0200	0.0847	0.0398	0.1864	**0.2559**	0.0630	0.0135	0.0835	**0.2090**	0.1500

Bold values are statistically significant with P <0.05.

**Fig 4:**
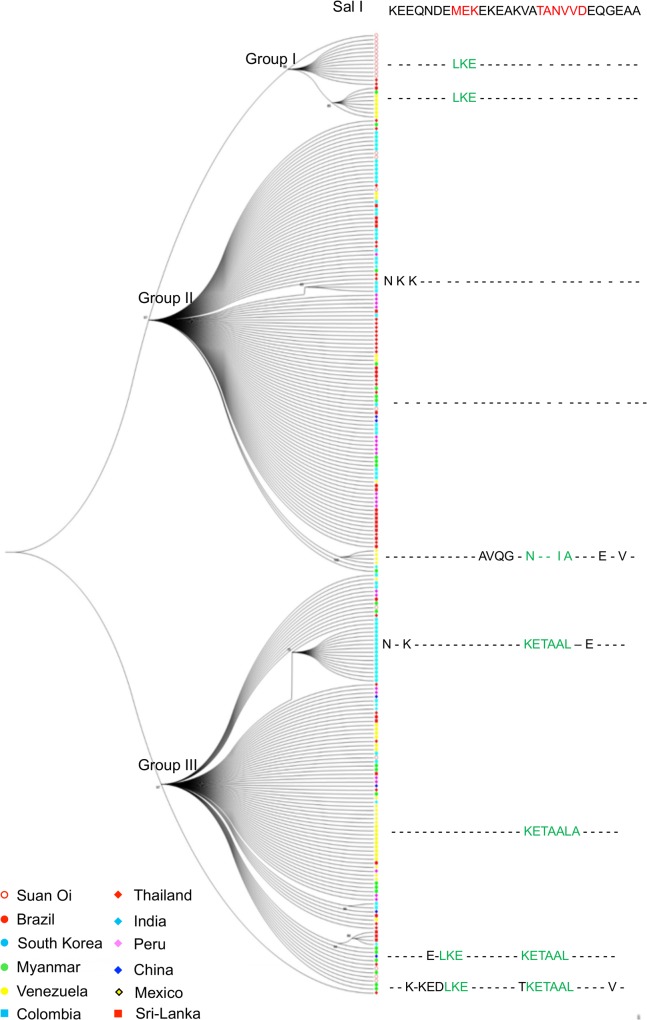
Maximum likelihood phylogeny of *PvMSP3*α block II DNA sequences. An unrooted phylogeny of the 237 *PvMSP3*α block II sequences was inferred with maximum likelihood using Tamura and Nei’s model of nucleotide substitution implemented in MEGA6. Bootstrapping was performed with 500 replicates and tree was condensed using 75% bootstrap as a threshold. The label of each sequence is color coded corresponding to the country of origin. Three major clusters were identified as group I, II and III and several sub-clusters were observed within each group. Consensus protein sequences generated for each cluster/sub-cluster using 29 parsimony informative amino acid changes have been shown. Salvador I sequence has been shown as a reference. Amino acids highlighted in red are indicating wild alleles of structural motif I and II while amino acids in green are the mutated alleles. Dashes '-' are representing amino acids that are similar in all the clusters.

Absence of population structuring was further supported by ML analysis of the 237 *PvMSP3α* block II sequences. Phylogenetic relations between isolates revealed three robust clusters (with bootstrap value more than 75%) based on sequence variations. Group I included 21 sequences from only four countries (Thailand, Myanmar, Brazil and Venezuela), whereas Group II and III included 112 and 104 sequences from all the 11 populations, respectively (Fig 4). High-frequency variations in the regions of two structural motifs mainly determined the pattern of clustering, though other variations defined few sequences that formed sub-clusters within the three major groups (Fig 4). Group I sequences contained the Sal I allele (wild type) in both motifs I and II (LSKLEE and TAANVVKD). In comparison, group II showed mutated allele only in motif I (MSELEK and TAANVVKD), whereas group III contained both alleles of motif I (LSKLEE/MSELEK) and only mutated allele in motif II (KEATAAKL).

### Selection and recombination

The rate of synonymous substitutions was found significantly higher than the rate of non-synonymous substitutions in worldwide sequences as well as in each population (data not shown), which indicates purifying/negative selection on block II. This observation was further supported by the significant negative values of Tajima’s D and Fu and Li’s F* tests observed in worldwide sequences. The Tajima’s D and Fu and Li’s F* values were positive in eight populations, but the deviation was significant only in Peru and Venezuela populations (Table 2). Interestingly, window plot analysis of Tajima’s D and Fu and Li’s F* in each population observed significantly positive values in a small region (from 582–591) that covers structural motif II (Fig 3B and 3C). This was further supported by performing the comparative analysis of structural motif II region (24bp) and rest of the block II (672bp) from all 237 sequences. Block II sequences (672 bp) showed highly significant negative values of Tajima's D (-1.9064, P<0.05) and Fu & Li's F (-7.1944, P<0.02), while motif II sequences (24 bp) showed significant positive values of Tajima's D (2.7062, P<0.05) and Fu & Li's F (1.3084, P>0.05). These observations suggest the influence of purifying selection on the entire block II possibly due to structural constraint forced by alanine heptad repeats, whereas a small region containing motif II might have experienced balancing selection. Interestingly, previous *in vitro* studies have localized a B-cell epitope in motif II (IDEB database; http://www.iedb.org/). Moreover, *in silico* B Cell Epitope Prediction server [57] also predicted both alleles of motif II as B cell epitopes with >75% specificity.

Intragenic recombination has been repeatedly reported as a prominent evolutionary force in maintaining genetic diversity in *PvMSP3α*. Since recombination rates estimated by different methods vary with the number of sequences, rate of recombination, and the number of recombination sites, we analyzed all 237 sequences together using five different tests for detecting recombination events. Among phylogenetic approaches of recombination detection, RDP, GENECONV and GARD tests failed to identify any breakpoints, while MaxChi identified one recombination breakpoint. In contrast to these phylogenetic approaches, 13 recombination events were identified by population genetics based estimator using DnaSP. We assumed that singletons might have influenced the DnaSP results, but singleton-free data (replaced singletons with major alleles) produced similar results. Recombination is also evident by eye, since any combinations of dimorphic alleles of the two structural motifs were observed among 237 sequences, implying that recombination in block II has taken place. This was further supported by the recombination event observed by DnaSP in the region between the two motifs (data not shown). Moreover, the estimated recombination rate (*ρ* = 0.06) was higher than mutation rate (*θ* = 0.0375) leading to a *ρ*/*θ* ratio of 1.6. Recombination to mutation ratio exceeding 1 signifies that the recombination is more prevalent in the dataset than mutation.

## Discussion

Analyzing the diversity of gene encoding antigens and the mechanisms involved in the maintenance of such variation is a necessary step for prioritizing vaccine candidates and monitoring their efficacy [33]. The importance of this can be illustrated by studies in *P*. *falciparum* that identified considerable diversity in the haplotypes used for designing MSP3-LSP and MSP1_19_ vaccines [58–61]. Significant genetic diversity was assumed to be one of the plausible reasons for the failures of these vaccines in clinical trials. The issue is particularly important in *P*. *vivax* since its antigens are still understudied and it has been observed that many genes encoding vaccine candidates in *P*. *vivax* show different patterns than *P*. *falciparum* [22, 61–64]. The block II of *PvMSP3α*, being relatively conserved with restricted variations has been proposed as a good vaccine candidate, but defining immunological relevance of the region is required. We analyzed *PvMSP3α* block II in 11 *P*. *vivax* endemic countries worldwide to highlight the extent and distribution of polymorphisms and the potential mechanisms generating these variation patterns. Block II was found less diverse compared to other vaccine candidate genes and many mutations were singletons. The pattern of variation was extensively shared by diverse *P*. *vivax* populations suggesting functional/structural constraint on block II, however, each population maintained different allelic forms of block II.

Though a large number of SNPs were observed in *PvMSP3α* block II of worldwide *P*. *vivax* populations, 66% (104/158) of them were singletons. Singletons and low-frequency alleles are generally excluded from diversity analysis of vaccine candidate genes [43] to avoid sequencing and/or PCR artifacts, especially for data retrieved from public databases when sequence accuracy cannot be confirmed. It is worth noting that singletons are expected in a population under expansion, a pattern that has been found in other studies [56, 65]. Alternatively, negative selection on functional genes also increases singletons [66]. The nucleotide diversity of worldwide samples was 0.019 with slight variations among 11 diverse populations (ranging from 0.015 to 0.023). The nucleotide diversity of *PvMSP3α* block II in each population was found lower than that of the full-length *PvMSP3α* gene [24, 27] as well as many other merozoite surface proteins analyzed in *P*. *vivax* populations, *e*.*g*., *PvMSP1 (*θπ = 0.027) [67], *PvMSP7C*, *PvMSP7H and PvMSP7I* (θπ = 0.057, 0.0357 & 0.043) [68], *PvMSP3β* (θπ = 0.0367) [23] and *PvMSP5* (θπ = 0.0375) [8]. However, the level of diversity in block II was high as compared to *PvMSP8* and *PvMSP10* (θπ = 0.0033 & 0.0022) [69]. This reflects that *PvMSP3α* block II is relatively conserved among many merozoite surface proteins in *P*. *vivax* populations. Additionally, nucleotide diversity in block II showed a non-random pattern, as peaks of nucleotide diversity were restricted to certain regions of the block II. A similar trend was observed when samples from different geographical regions were analyzed, suggesting of functional/structural constraint, since block II is rich in alanine heptad repeats that are predicted to form coiled coil structures possibly needed for the functioning of *PvMSP3α* gene [26].

Clustering analysis of block II sequences revealed a general lack of geographic structure. However, three robust clusters, each comprising of mixture of sequences from diverse populations were also observed. Moreover, highly frequent non-synonymous SNP-based haplotypes were shared by multiple populations irrespective of their geographical locations, which suggest either extensive gene flow between populations, or independent convergence of variations due to their functional/structural importance. The phylogenetic grouping was found to be based on the type of sequence variations especially influenced by the presence/absence of dimorphic alleles of two structural motifs, suggesting of selective pressure on these motifs.

Antigenic genes are generally expected to be under diversifying/balancing selection, genes under such selective pressure could show lower levels of genetic differentiation between populations than the one expected by genetic drift alone. Accordingly, the overall F_ST_ value in worldwide samples was 0.09 with the highest estimate was observed between Thailand and Mexico as 0.2559, P<0.05. Moreover, an F_ST_ value of 0.012 between Asian (n = 139 sequences) and American (n = 98) samples analyzed in this study is remarkably low as compared to the F_ST_ estimates previously observed between Asian and American *P*. *vivax* populations using mitochondrial DNA (F_ST_ = 0.15–0.50) [56] and silent SNPs (F_ST_ = 0.228) [70]. This might be due to shared non-synonymous variations between populations which tend to maintain low F_ST_ values [71], a pattern consistent with balancing selection, but these results need to be interpreted with caution given our small sample sizes.

Though recombination seems to play an important role in generating new genetic variants, polymorphisms in block II are largely clustered in particular regions. Moreover, the pattern of amino acid changes is shared by diverse populations, possibly due to purifying selection that might be acting on alanine heptad repeats for structural conservation. However, a small region in block II encoding motif II showed evidences of balancing selection. Particularly, two alleles of this motif seem to maintain intermediate frequencies in the study populations. This motif might be involved in host pathogen interaction since one of the epitope identified by *in vitro* studies is localized in this region. Moreover, both alleles were predicted as B cell epitopes with >75% specificity. Blocking this region might prevent merozoite invasion of reticulocytes, and inclusion of both alleles in a vaccine design might be able to induce immune responses recognizing both alleles. However, immunological studies are required to assess the immunogenicity and protection-inducing capability of both alleles in natural *P*. *vivax* infections in diverse population backgrounds.

Conserved pattern of amino-acid variations in block II of *PvMSP3α* compared to the full-length *PvMSP3α* as well as many other merozoite surface proteins provides a strong support for vaccine development based on block II. An important observation is the common and extensively shared pattern of polymorphisms among diverse populations, which increase the possibility of formulating vaccines effective against worldwide *P*. *vivax* populations. Though conserved regions in malaria antigens are generally not highly immunogenic and protective [72, 73], antibodies against block II have been significantly associated with protection against clinical *P*. *vivax* infections [28]. This study also identified a B cell epitope in a small region in block II, which was also predicted to be under immune selection, suggesting that it is probably involved in direct interactions with the host cells. Noticeably, only two prominent alleles were observed in this epitope worldwide and both of them showed equivalent specificity as a B cell epitope. Functional studies are needed to determine the immunogenicity and protection ability of these small polypeptides against *P*. *vivax* infections.

## Supporting Information

S1 Table Accession numbers of *Plasmodium vivax* merozoite surface protein 3α (*PvMSP3α*) sequences retrieved from GenBank and Plasmodb. (DOC)
