# Supplementary material for: Molecular Evolution of PvMSP3α Block II in Plasmodium vivax from Diverse Geographic Origins
Source: PLoS One. 2015 Aug 12;10(8):e0135396. doi: 10.1371/journal.pone.0135396 (PMC4534382; doi:10.1371/journal.pone.0135396)
Supplement: S1 Table — (DOC) [file pone.0135396.s001.doc]

**S1 Table. Accession numbers of *Plasmodium vivax* Merozoite Surface Protein 3α (*PvMSP3α*) sequences retrieved from GenBank and Plasmodb.**

| **Brazil (12):** |
| --- |
| Brazil01, Brazil22, Brazil30, Brazil32, Brazil33, BrazilI, AF491956, AF491949, AF491945 - AF491947, SAL1 |
| **China (6):** |
| China_LZCH-13, China_LZCH-20, China_LZCH-4, China_NB-15, China_NB-16, China_NB-17 |
| **Columbia (22)** |
| Columbia_30101099036, Columbia_30101099040, Columbia_30102100437, Columbia_30102100438-A, Columbia_30102100438-B, Columbia_30102100439, Columbia_30102100440, Columbia_30102100441-B, Columbia_30102100445, Columbia_30102100446, Columbia_30102100448, Columbia_30102100485, Columbia_30102100486, Columbia_30102100488, Columbia_30102100489, Columbia_30102100490, Columbia_30102100491, Columbia_30102100504, Columbia_30103103280, Columbia_30111110015, Columbia_30111110020, Columbia_30111110026 |
| **India (6):** |
| IndiaVII, HQ328853 - HQ328855, AF491957, KC935446 |
| **Mexico (15):** |
| Mexico_1086-A, Mexico_118-A, Mexico_161-04, Mexico_165-A, Mexico_203-04, Mexico_21-A, Mexico_267-A, Mexico_32-E-03, Mexico_330-A, Mexico_55-03, Mexico_566-A, Mexico_63-08, Mexico_760-A, Mexico_938-A, Mexico_980-A |
| **Peru (23)** |
| Peru00622, Peru00692, Peru00699, Peru06, Peru07, Peru08, Peru1008, Peru2025, Peru257, Peru259, Peru260, Peru262, Peru3043_D0, Peru3133, Peru3136, Peru3232, Peru3270_D43, Peru3270_D59, Peru3323, Peru4023, Peru858, Peru872, Peru99622 |
| **Sri-Lanka (17)** |
| GU175269 - GU175277, GU175279 - GU175285, AF491961 |
| **Myanmar (25)** |
| EU430576 - EU430600 |
| **South Korea (33)** |
| JQ317283, JQ317284, JQ317286 - JQ317289, EF204144 - EF204152, EF204154 - EF204171 |
| **Venezuela (26)** |
| AJ864941- AJ864942, AJ864944 - AJ864967 |
| **Thailand (32)** |
| AF491962, AY833010 - AY833026, Thailand_VKBT-100, Thailand_VKBT-101, Thailand_VKBT-106, Thailand_VKBT-72, Thailand_VKBT-94, Thailand_VKBT-95, Thailand_VKBT-98, Thailand_VKBT-99, Thailand_VKTS-36, Thailand_VKTS-37, Thailand_VKTS-39, Thailand_VKTS-45, Thailand_VKTS-52, KC935442.1 |
| **Suan Oi (20)** |
| KR902511 - KR902530 |
